# Lévy patterns in seabirds are multifaceted describing both spatial and temporal patterning

**DOI:** 10.1186/s12983-016-0160-2

**Published:** 2016-06-29

**Authors:** Andrew M. Reynolds, Vitor H. Paiva, Jacopo G. Cecere, Stefano Focardi

**Affiliations:** Rothamsted Research, Harpenden, AL5 2JQ UK; MARE – Marine and Environmental Sciences Centre, Department of Life Sciences, University of Coimbra, Coimbra, 3004-517 Portugal; ISPRA, Ozzano dell’Emilia, 40064 Italy; LIPU, Conservation Department, Parma, 43100 Italy; ISC-CNR, Sesto Fiorentino, 50019 Italy

**Keywords:** Foraging, Lévy statistics, Power-laws, *Procellariiformes*, Shearwaters

## Abstract

**Background:**

The flight patterns of albatrosses and shearwaters have become a touchstone for much of Lévy flight research, spawning an extensive field of enquiry. There is now compelling evidence that the flight patterns of these seabirds would have been appreciated by Paul Lévy, the mathematician after whom Lévy flights are named. Here we show that Lévy patterns (here taken to mean spatial or temporal patterns characterized by distributions with power-law tails) are, in fact, multifaceted in shearwaters being evident in both spatial and temporal patterns of activity.

**Results:**

We tested for Lévy patterns in the at-sea behaviours of two species of shearwater breeding in the North Atlantic Ocean (*Calonectris borealis*) and the Mediterranean sea (*C. diomedea*) during their incubating and chick-provisioning periods. We found that distributions of flight durations, on/in water durations and inter-dive time-intervals have power-law tails and so bear the hallmarks of Lévy patterns.

**Conclusions:**

The occurrence of these statistical laws is remarkable given that bird behaviours are strongly shaped by an individual’s motivational state and by complex environmental interactions. Our observations could take Lévy patterns as models of animal behaviour to a new level by going beyond the characterisation of spatial movements to characterise how different behaviours are interwoven throughout daily animal life.

## Background

A key objective of the emerging discipline of ‘Movement Ecology’ is to characterize how resting and active periods are interwoven throughout an animals’ daily life [1]. The search for statistical rules describing such complex patterns of behaviours remains a difficult and often elusive goal. But the identification of such rules is crucial for the accurate forecasting of animal behaviours and decision making, and is a crucial first step in the identification of the underlying generative processes which will provide a foundation for understanding and a scientific basis for extrapolation [2].

Given the long standing assumption of scale-dependency of ecological patterns [2], it is perhaps natural to presuppose that animal activity patterns will be Poisson – one of the most important, most studied and frequently encountered random process. If this were true then the time intervals between two consecutive actions by the same individual, called the waiting or inter-event time, would be exponentially distributed with a characteristic scale. And as a consequence, consecutive actions would be expected to follow each other at relatively regular time intervals and very long waiting times would be forbidden. This, however, is at variance with recent empirical studies which have revealed that the timing patterns of animal behaviours can deviate significantly from the Poisson/exponential prediction, as waiting times and inter-event times can be better represented by heavy tailed distributions which allow for very long durations and which in principle are scale free [3–5].

Wearmouth et al. [3] reported that the waiting times of marine sit-and-wait ambush predators are power-law distributed across a broad set of scales. Such behaviours have also been observed in more mobile little penguins (*Eudyptula minor*) [4]. Reynolds et al. [5] subsequently reported that the pause and movement durations of a variety of invertebrates are also power-law distributed across a broad set of scales when individuals are exposed to minimal external cues. The occurrence of these similar statistical laws is remarkable given that behaviours are strongly shaped by motivational states and environmental interactions. These studies raise the intriguing possibility that animals use evolutionarily encoded priority-based queuing mechanisms to decide between competing activities as first suggested by Barabási [6] and then demonstrated theoretically by Reynolds [7]. This has the potential to open a new window onto animal behaviour. But it remains to be seen whether or not these statistical laws are pervasive, operating in more complex settings where there are multiple stimuli and strong environmental interactions. If they do then they would mirror Lévy walks – a popular model of spatial movements.

Lévy walks also known as Lévy flights in the biological literature comprise clusters of many short steps with longer steps between them. This pattern is repeated across all scales with the resultant clusters creating fractal patterns that have no characteristic scale. The hallmark of a Lévy flight is a distribution of step lengths with a heavy power-law tail; *p*(*l*) ~ *l*^− *μ*^ with 1 < *μ* ≤ 3, where *l* is the step-length and *μ* is the power-law (Lévy) exponent (here ‘~’ means ‘distributed as’). Shorter movements can be distributed differently. But irrespective of how the short movements are distributed, net displacements tend to Lévy stable distributions as the number of steps grows by virtue of a generalised central limit theorem due to Gnedenko and Kolmogorov [8]. It is this convergence to Lévy stable distributions which justifies calling movement patterns with heavy-tailed step-length distributions, Lévy flights. On the other hand, when step-length distributions have thin tails, net displacements eventually become Gaussian distributed by virtue of the central limit theorem. Note, however, that in the mathematics and physics literature, a distinction is made between “Lévy flights” where steps are made instantaneously and “Lévy walks” where speed is constant (so that the duration of a step is proportional to its length). Here following the convention in the biological literature we use the term “Lévy flight” as a proxy for a “Lévy walk”. Interest in Lévy flights as models of spatial movements exploded after it was reported that they can be discerned in the flight patterns of wandering albatrosses (*Diomedea exulans*) [9]. It subsequently became apparent that this study and many that followed were seriously flawed through the use of inappropriate statistical techniques and in some cases through misinterpretations of the data [10, 11]. These flawed early studies continue to cast a long shadow over Lévy flight research and in some quarters Lévy flight modelling is met with fierce resistance [12]. Nonetheless, there is now seemingly compelling evidence that many organisms have movement patterns resembling Lévy flights. Lévy flight movement patterns have, for instance, been observed to some extent in bacteria *E. coli* [13, 14], T cells [15], a diverse range of aquatic marine predator [16–18], mussels [19], mud snails [20, 21], honeybees [22], shearwaters [23, 24], human hunter-gatherers [25] and have even been observed in trace fossils – the oldest records of animal movement patterns [26].

In many cases these movement patterns have been interpreted within the context of the Lévy flight foraging hypothesis which posits that because Lévy flights can optimize search efficiencies, natural selection should have led to adaptations for Lévy flight foraging [27]. Their occurrence can, however, be understood more generally as arising naturally from innate behaviours and environmental interactions [28]; interpretations which dispense with the heated arguments about whether or not Lévy movements are in some sense optimal [12].

There is now strong evidence that wandering albatrosses do, after all, perform Lévy flights [29, 30]. These analyses do, in fact, suggest that Lévy patterns (i.e., processes characterized by distributions with heavy power-law tails) proliferate in wandering albatrosses describing both spatial and temporal patterning [29, 30]. Humphries et al. [30] found the hallmarks of Lévy flights in flight pattern data. Humphries et al. [29] reported that the flight durations of the wandering albatrosses are power-law distributed, under specific ecological circumstances of scarce food availability. This has resonance with the statistical rules uncovered by Wearmouth et al. [3], and by Reynolds et al. [4, 5] and suggests that this rule can operate in complex, stimuli-rich settings. In other words, the statistical rule is not indicative of some kind of ‘idling mode’ which when acted on by environmental cues, produces the movements with characteristic scales sometimes observed in nature.

Here we test this possibility by examining how flights and bouts of on/in water activities are interwoven into the daily lives of two species of shearwaters which like wandering albatrosses are *Procellariiformes* and like wandering albatrosses have Lévy flight movement patterns [24, 25]. In the discussion we identify putative, biologically plausible generative mechanisms for the patterns we observe.

## Results

**Fig. 1:**
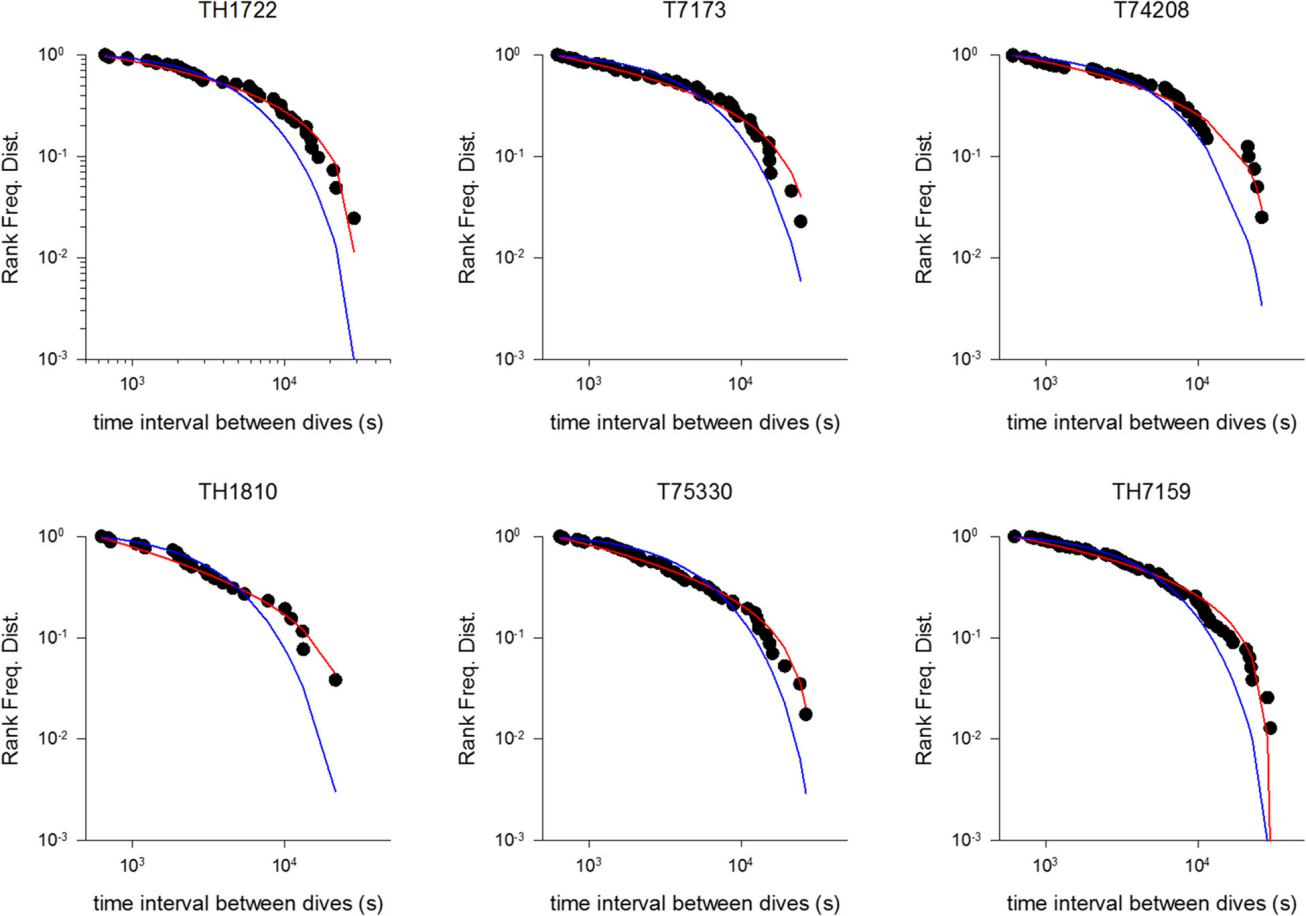
Example rank frequency plots of the time intervals between consecutive daytime dives for incubating *C. diomedea* breeding in the Mediterranean (Linosa) (●). Shown for comparisons are the best fit truncated power-laws (*red*) and best fit exponentials (*blue*)

**Fig. 2:**
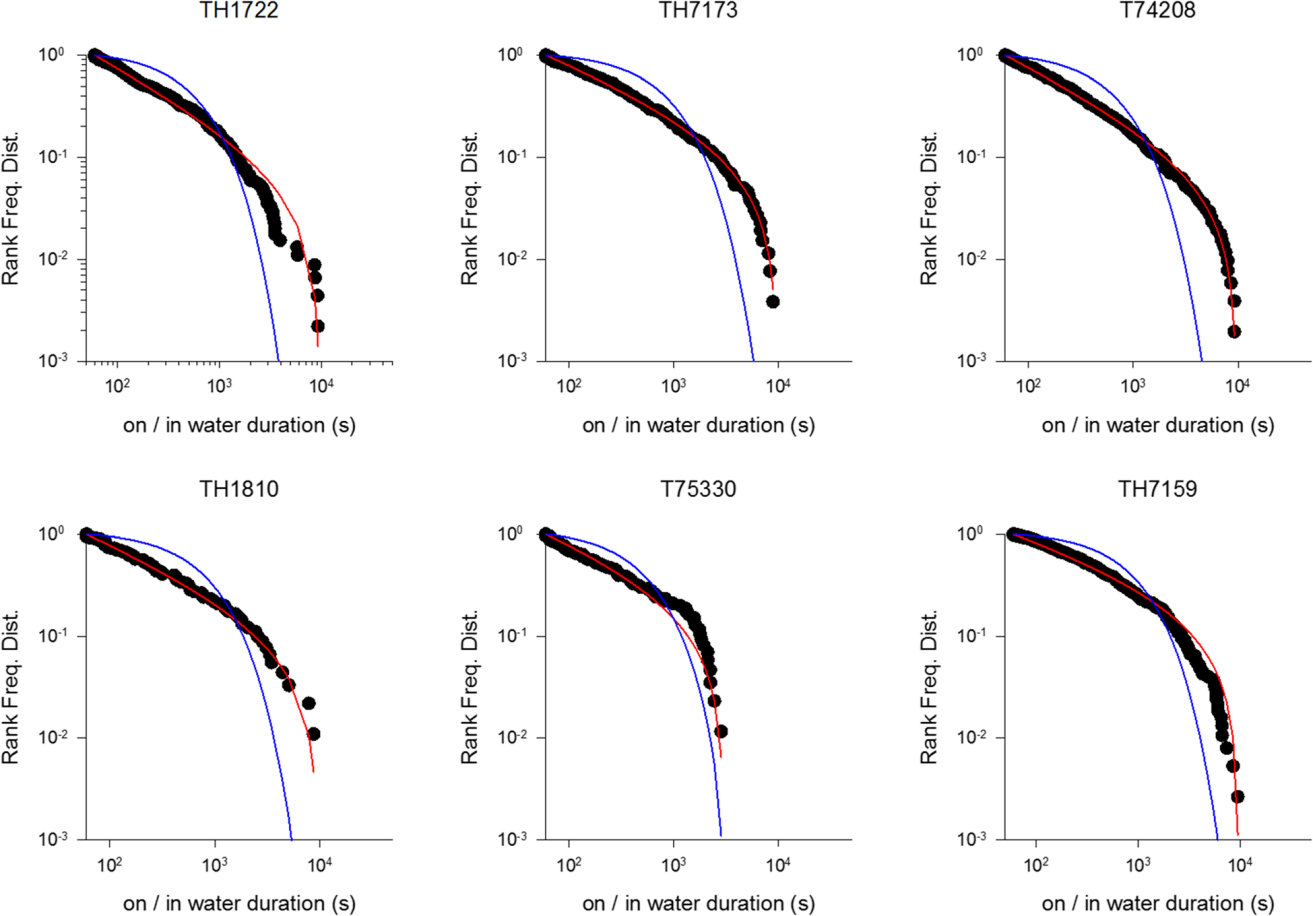
Example rank frequency plots of daytime on/in water durations for *C. diomedea* breeding in the Mediterranean (Linosa) (●). Shown for comparisons are the best fit truncated power-laws (*red*) and best fit exponentials (*blue*)

**Fig. 3:**
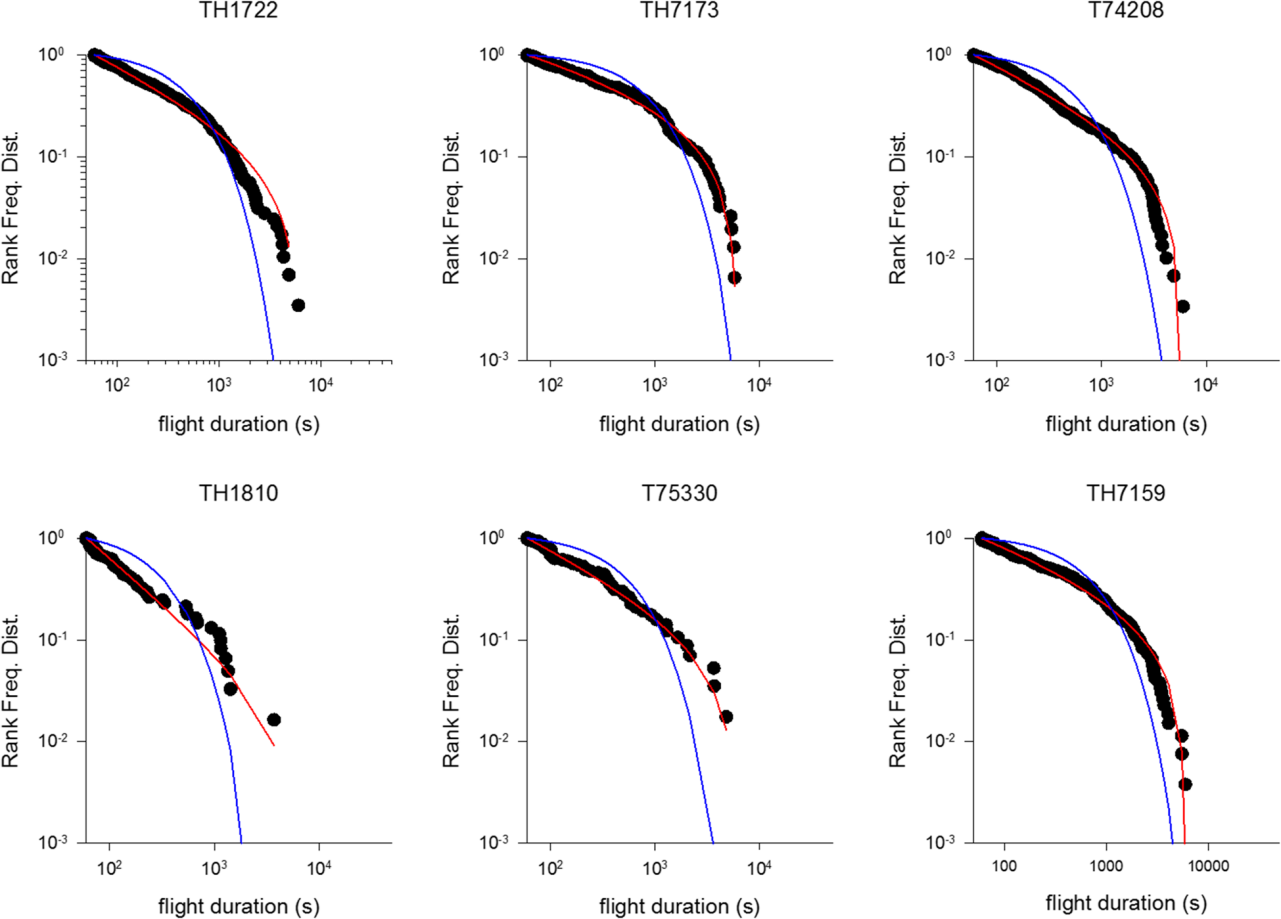
Example rank frequency plots of the durations of daytime flights for incubating *C. diomedea* breeding in the Mediterranean (Linosa) (●). Shown for comparisons are the best fit truncated power-laws (*red*) and best fit exponentials (*blue*)

We found evidence of the hallmark of Lévy patterns in the flight durations, on/in water durations and the inter-dive time-intervals in almost all of the birds analysed. All three quantities are to good approximation power-law distributed in Scopoli’s shearwater (Ss) *Calonectris diomedea* breeding in the Mediterranean (Linosa) (Figs. 1, 2 and 3, Tables 1 and 2). In most cases power-laws provide better fits to our data than do simple exponentials and in all cases power-laws are favoured over exponentially-truncated power-laws. The exponentially-truncated power-law fits typically reduce to truncated power-laws because the maximum likelihood estimates for the exponential decay rate, λ_2_, are usually zero (or so small that the exponential decay is negligibly small over the range of observed scales) and for this reason are not displayed. These fits are also penalized by the Akaike information criterion by virtue of having 2 parameters (μ and λ_2_) rather than 1 parameter (μ). Power-law scaling typically extends over 2 or more decades. This fulfils Stumpf and Porter’s [31] ‘rule of thumb’ for the reliable detection of a power-law.

**Table 1:** Akaike weights for truncated power-laws, maximum likelihood estimates for power-law exponents and *p*-values quantifying the absolute goodness-of-fit of the power-law across chick-provisioning *C. diomedea* breeding in the Mediterranean (Linosa). Estimates for the power-law exponents are only given when the evidence for a power-law is strong

Bird (individual numbered bands)	Akaike weight for a power-law;	MLE for *μ*;	*p*-value
	On/In water, Flying and Diving	On/In water, Flying and Diving	On/In water, Flying and Diving
TH1826	1.00, 1.00, 0.65	1.47, 1.39,1.40	0.81, 0.29, 0.78
TH8446	1.00, 0.84, 0.80	1.25, 1.53, 1.00	0.87,0.19, 0.20
T74208	1.00, 0.28, 0.67	1.74, 1.60, 1.00	0.55,0.79, 0.59
T73749	1.00, 1.00, 0.09	1.30, 1.51, -	0.65,0.95, 0.29
TH7179	1.00, 1.00, 0.59	1.35, 1.36, 1.00	0.43,0.38, 0.71
All individuals	1.00,1.00, 0.99	1.43,1.37,1.08	

Note that the combined Akaike weight for the alternative models (i.e., for either the exponentially-truncated power-law or the exponential being the better model of our data) is 1 – Akaike weight for a truncated power-law

**Table 2:** Akaike weights for truncated power-laws, maximum likelihood estimates for power-law exponents and *p*-values quantifying the absolute goodness-of-fit of the power-law for incubating *C. diomedea* breeding in the Mediterranean (Linosa)

Bird (individual numbered bands)	Akaike weight for a power-law;	MLE for *μ*;	*p*-value
	On/In water, Flying and Diving	On/In water, Flying and Diving	On/In water, Flying and Diving
TH7159	1.00, 1.00, 0.92	1.30, 1.34, 1.06	0.27, 0.28, 0.86
TH7174	1.00, 1.00, 0.05	1.54, 1.44, 1.13	0.58, 0.84, 0.77
TH7160	1.00, 1.00 1.00	1.50, 1.42, 1.01	0.95, 0.00, 0.91
TH7021	1.00, 1.00, 1.00	1.45, 1.39, 1.13	0.95, 0.96, 0.77
TH1722	1.00, 1.00, 0.93	1.55, 1.48, 1.00	0.15, 0.51, 0.49
TH7172	1.00, 1.00, 0.05	1.65, 1.48, 1.01	0.85, 0.46, 0.78
TH7173	1.00, 1.00, 0.97	1.41, 1.23, 1.11	0.67, 0.90, 0.08
T75330	1.00, 1.00, 0.94	1.40, 1.51, 1.19	0.96, 0.29, 0.16
T73735	1.00,0.61, 0.72	1.67, 1.40, 1.26	0.34, 0.38, 0.99
TH7203	1.00, 1.00, 0.90	1.21, 1.27, 1.14	0.05, 0.26, 0.42
TH1810	1.00, 1.00, 0.83	1.44, 1.88, 1.32	0.15, 0.60, 0.02
TH4317	1.00, 1.00, 1.00	1.51, 1.46, 1.06	0.03, 0.99, 0.14
TH8443	1.00, 1.00, 1.00	1.56, 1.67, 1.07	0.95,0.66, 0.37
T8442	1.00, 1.00, 1.00	1.48, 1.42, 1.28	0.71, 0.21, 0.95

Note that the combined Akaike weight for the alternative models (i.e., for either the exponentially-truncated power-law or the exponential being the better model of our data) is 1 – Akaike weight for a truncated power-law

**Fig. 4:**
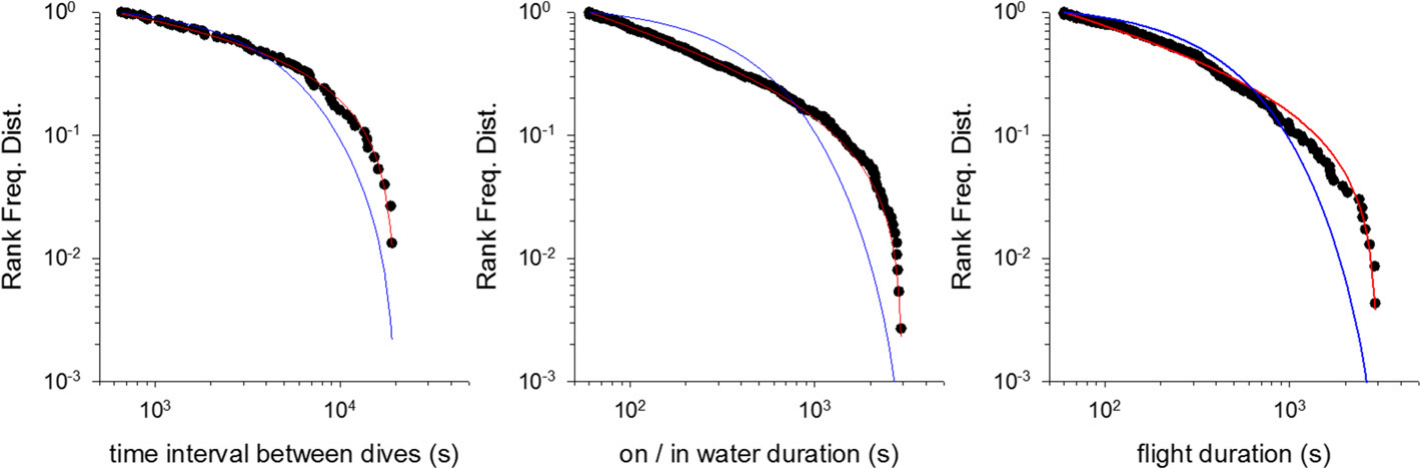
Example rank frequency plots of the time-intervals between dives, on/in water durations and flight durations for chick-provisioning *C. diomedea* breeding in the Mediterranean (Linosa) (●). Daytime data has been pooled for 5 individuals. Shown for comparisons are the best fit truncated power-laws (*red*) and best fit exponentials (*blue*)

**Fig. 5:**
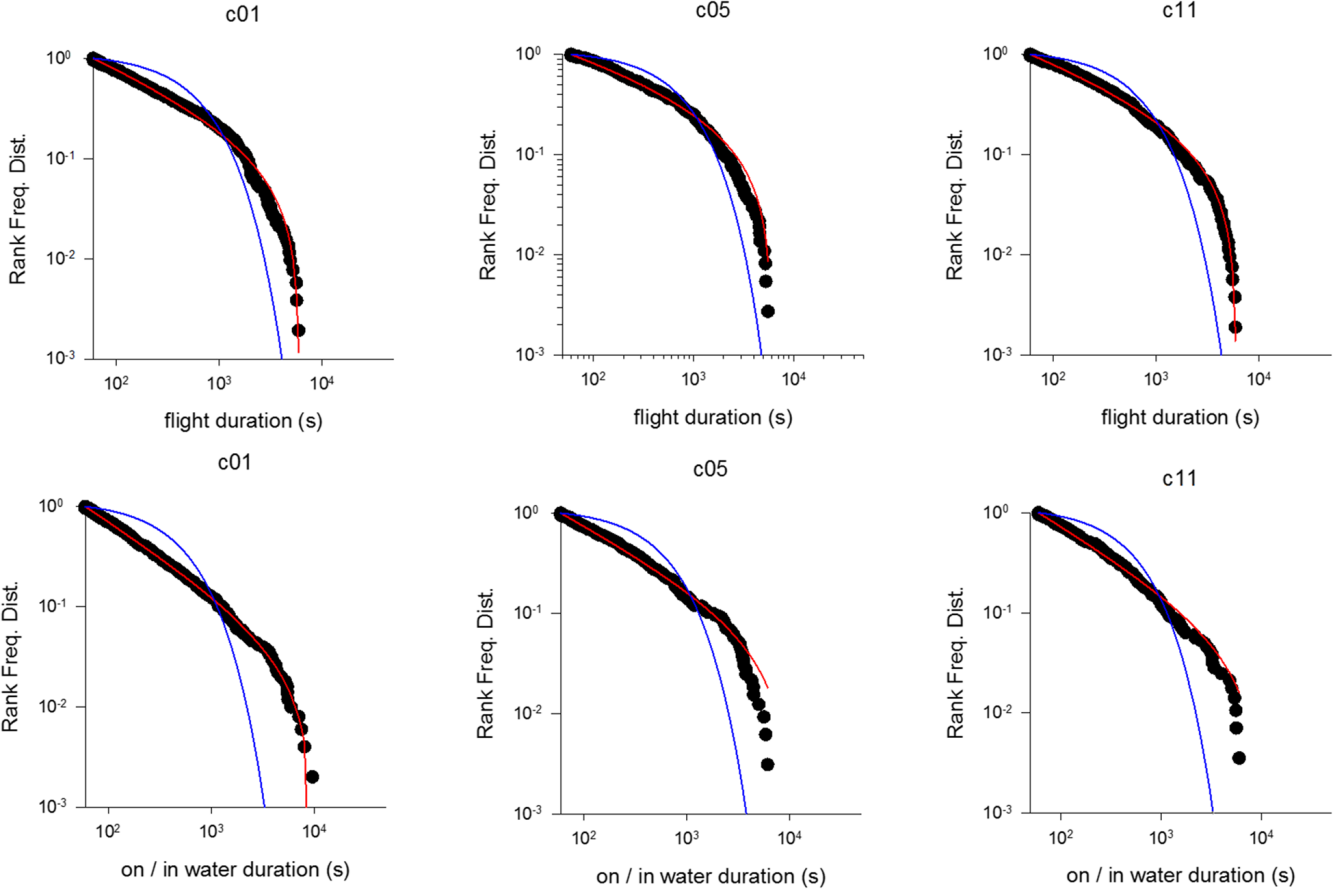
Example rank frequency plots of the flight durations and daytime in/on water durations for incubating *C. borealis* breeding in the North Atlantic (Corvo) (●). Shown for comparisons are the best fit truncated power-laws (*red*) and best fit exponentials (*blue*)

**Fig. 6:**
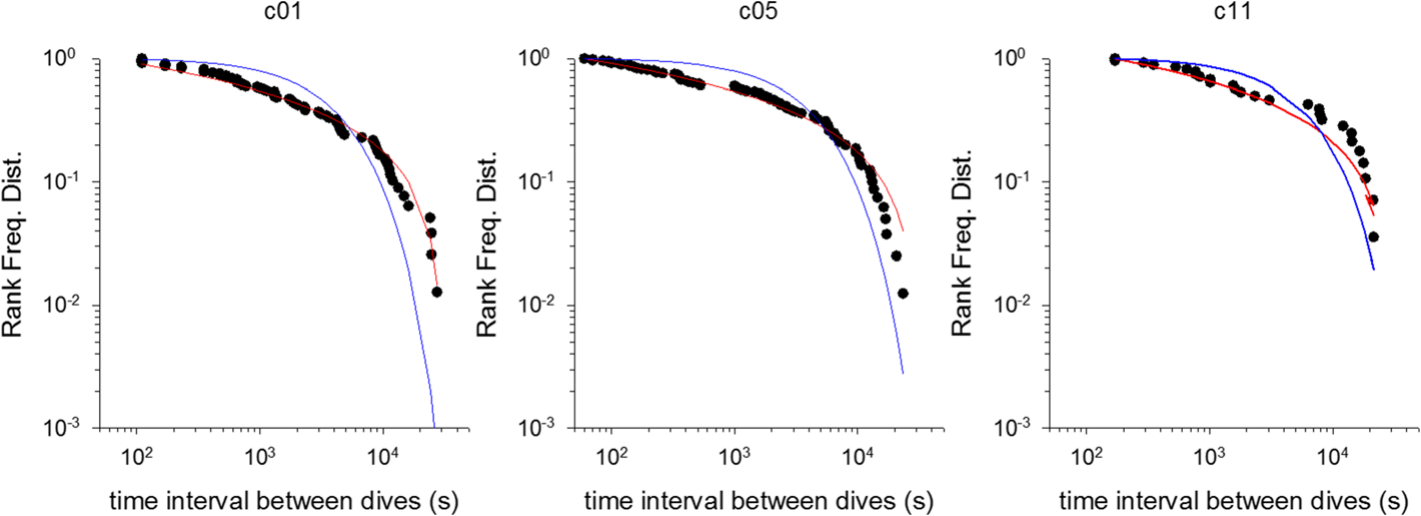
Example rank frequency plots of the time intervals between consecutive daytime dives for incubating *C. borealis* breeding in the North Atlantic (Corvo) (●). Shown for comparisons are the best fit truncated power-laws (*red*) and best fit exponentials (*blue*)

For some individuals the power-law fitting very closely matches our observations (Figs. 1, 2 and 3). This is most evident in the long incubation flights where data are plentiful, but less so in the chick-rearing flights. Nonetheless, power-law scaling in the chick rearing flights becomes manifest when the data for all individuals are pooled (Fig. 4). Power-law scaling is not specific to the Ss as it is also evident in two populations of Cory’s shearwater (Cs) *C. borealis* breeding in the North Atlantic (Corvo, Berlenga) (Figs. 5-6, Table 3). Results are only given for incubating Cs because there are insufficient data in individual chick rearing flights to distinguish reliably between power-laws and exponentials in this species.

**Table 3:** Akaike weights for truncated power-laws, maximum likelihood estimates for power-law exponents and *p*-values quantifying the absolute goodness-of-fit of the power-law for incubating *C. borealis* breeding in the North Atlantic (Corvo and Berlenga). Estimates for the power-law exponents are only given when the evidence for a power-law is strong

Bird (individual numbered bands)	Akaike weight for a power-law;	MLE for *μ*;	*p*-value
	On/In water, Flying and Diving	On/In water, Flying and Diving	On/In water, Flying and Diving
B01-1	0.81, 0.09, 0.10	1.11, -, -	0.62, 0.36, 0.41
B03	1.00, 1.00, 1.00	1.25, 1.22, 1.22	0.66, 0.87, 0.10
B04	1.00, 1.00, 1.00	1.46, 1.61, 1.30	0.72, 0.62, 0.26
B05	1.00, 1.00, 1.00	1.55, 1.34, 1.07	0.06, 0.46, 0.22
B06	1.00, 1.00, 0.06	1.74, 1.39, -	0.19, 0.01, 0.63
B07	0.06, 0.07, 0.12	-, -, -	0.03, 0.62, 0.09
B08-1	0.61, 1.00, 0.91	1.41, 1.41, 1.00	0.01, 0.31, 0.81
C01	1.00, 1.00, 1.00	1.44, 1.67, 1.11	0.85, 0.19, 0.50
C02-1	1.00, 0.00, 1.00	1.44, -, 1.31	0.49, 0.82, 0.57
C03	0.03, 1.00, 0.01	-, 1.15, 1.43	0.26, 0.12, 0.19
C04-1	1.00, 1.00, 0.42	1.45, 1.32, 0.98	0.51, 0.77, 0.69
C05	1.00, 1.00, 1.00	1.28, 1.55, 1.12	0.07, 0.55, 0.71
C06	0.88, 1.00, 0.74	1.22, 1.46, 1.09	0.53, 0.11, 0.92
C10-1	0.44, 0.54, 1.00	-, -, 1.01	0.42, 0.42, 0.15
C11	1.00, 1.00, 0.93	1.37, 1.60, 0.96	0.47, 0.38, 0.48

Note that the combined Akaike weight for the alternative models (i.e., for either the exponentially-truncated power-law or the exponential being the better model of our data) is 1 – Akaike weight for a truncated power-law

Results are summarised in Table 4. There is no significant between-colony difference for flying and on/in water times (Kruskal-Wallis test, *χ*^2^ = 1.09, *P* = 0.58 and *χ*^2^ = 3.3, *P* = 0.18. respectively) while Corvo appears to be characterised by a lower value of the Lévy exponent for waiting times between dives than the other two colonies (Kruskal-Wallis test, χ^2^ = 7.13, *P* = 0.03). The comparison of Lévy exponent values among the three categories of behaviour confirms that there are no significant differences between flying and on/in water times (Kruskal-Wallis test, *χ*^2^ = 0.03, *P* = 0.85), while waiting times between dives are different (Kruskal-Wallis test, *χ*^2^ = 44.2, *P* < 0.0001). Additionally, we found no evidence of a correlation between flight durations and subsequent on/in water durations. Longer-than-average flights were, for example, not followed by longer-than-average on/in water durations.

**Table 4:** Colony, effective sample size and average MLE estimates for On/In Water, Flying and Diving times

Colony	N	On/In Water ± std	Flying ± sd	Diving ± sd
Berlenga	7	1.42 ± 0.22	1.39 ± 0.14	1.15 ± 0.14
Corvo	8	1.37 ± 0.1	1.46 ± 0.19	1.02 ± 0.06
Linosa	14	1.48 ± 0.12	1.46 ± 0.16	1.13 ± 0.11

## Discussion

Initial reports that albatrosses have movement patterns resembling Lévy flights [9], although flawed [10, 11], led eventually to the realization that many organisms have movement patterns which can be approximated as Lévy flights [13–26]. This commonality in movement among taxonomically well-separated organism is guiding on-going research aimed at unravelling the underlying physiological and behavioural mechanisms which in turn will facilitate understanding and prediction [28]. Our analysis suggests that the utility of Lévy statistics has, in fact, not yet been fully realized because Lévy statistics can and do describe both spatial and temporal patterns of behaviour.

We found that Lévy patterns do, in fact, proliferate in shearwaters, a species closely related to the wandering albatross, describing not only their flight patterns [24] but also flight durations, on/in water durations and inter-dive time-intervals. It seems that research into biological Lévy patterns still has a lot to learn from flight behaviours of *Procellariiformes.* Our findings suggest that the current focus on Lévy flight movement patterns rather than on spatial-temporal patterns is too restrictive, and that the hotly debated Lévy flight foraging hypothesis needs to be revised.

**Fig. 7:**
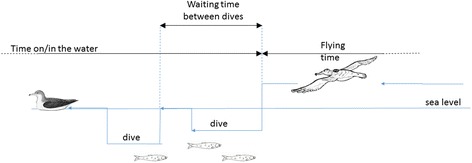
Illustrative sequence of flying, sitting on the water and diving events

There is no specific evidence of the underlying mechanisms which may generate the observed patterns, but we can try to develop working hypotheses to be used in forthcoming studies. Perhaps the most parsimonious explanation of our findings can be found in odour-cued responses, a possibility which resonates with that of shearwater’s flight patterns. These Lévy flight patterns are characterized by power-laws with exponents close to 3/2 and can be attributed to the birds assembling cognitive maps of wind-borne odours [24]. As a result of atmospheric turbulence these odour cues break up into packets (filaments) and disperse thereby acquiring an irregular patchily concentration distribution, and so are only present intermittently. When they are present with concentrations above the threshold of detection, the birds are with their map sense and make unidirectional flights. When the odours are absent the birds are without their navigational cues and attempt to re-establish contact with the map by turning. Turbulence theory predicts that the durations of time that the birds are with and without their map are both 3/2 power-law distributed (albeit with exponential truncation at very long times) [24]. When the birds travel with near constant speed these flight durations manifest themselves as the aforementioned Lévy flight patterns. Prey detection in shearwaters could also be odour-cued as evidenced in the wandering albatross (*Diomedea exulans*) [32]. High concentrations of dimethyl sulfide are indicative of high concentrations of phyto- and zoo-plankton and so potentially indicative of the presence of prey. And here it is also worth remarking that albatross vision is tuned to monitoring motion on the horizon (a distance of about 10 km for typical heights, ca. 8 m, above sea level where albatrosses fly), such as other bird activity, rather than spotting prey items from a distance [32]. Foraging shearwaters can be expected to fly until prey odours exceed some threshold whereupon they either dive into the water or land on the water and wait for their quarry (which is not necessarily there because turbulence can concentrate odours to give a false positive). The birds could then be expected to remain on the water until the prey odour falls back below the threshold whereupon they take to the air again. Turbulence theory then predicts that flight times and on/in water times will be 3/2 power-law distributed, mirroring the case of odour-cued navigation, i.e., navigational-odour cues trigger changes in flight heading whilst prey-odour cues trigger landings and take-offs. This effect may explain why the estimates for the power-law exponents are typically close to 3/2 (Table 4). It is consistent with the notion that during the breeding period birds are strongly motivated to find food, an activity usually performed during the day. It is therefore likely that landings (on the water) are triggered by detection of prey. The possibility that prey-odours will result in power-law distributions of inter-dive times was predicted nearly a decade ago [33, 34] when the study of Viswanathan et al. [9] was starting to garner widespread attention. The theory is supported by observations of bumblebee landing patterns and is applicable when prey (i.e., odour sources) are sparsely distributed so that the patchiness of odour concentrations is the result of turbulent processes rather than the prey distribution. When prey are abundant odour-cued dive patterns will tend to reflect the prey distribution and can be expected to be exponential. These speculations are consistent with the landing patterns of wandering albatrosses and black-browed albatrosses (*Thalassarche melanophrys*) [29]. Humphries et al. [30] reported that Lévy-flight landing patterns tended to occur over the deep shelf and oceanic waters where prey are sparsely distributed, whilst exponential landing patterns tended to occur over the shallow shelf and shelf edge habitats where prey are more abundant. Nonetheless, it is not clear to what extent, if any, prey-odour cues can account for the power-law distributions of inter-diving time-intervals which in contrast with flying and sitting durations are seascape (and so perhaps prey) dependent (Table 4). Analysis is not straightforward because between consecutive dives, birds can spend time both flying and sitting (floating) (Fig. 7).

Our findings resonate with those of Wearmouth et al. [3] who reported that the waiting times of sit-and-wait ambush predators are scale-free across a broad range of scales; a trait later found in more mobile penguins [4]. The occurrence of these statistical laws has been attributed to stochastic decision making [3, 4]. Stochastic decision-making processes was first proposed as an explanation for the power-law that seem to characterize how resting and active periods are interwoven throughout human daily life [6, 35–37]. But subsequent studies demonstrated that the power-law scaling of such inter-event distributions is apparent rather than actual and is, in fact, a consequence of circadian and weekly cycles [38, 39]. Non-Poisson inter-event distributions and stochastic decision-making processes are, nonetheless, gaining traction once again. They are, following Cole [40] and Martin [41], now finding application in the characterization and modelling of animal activities [3, 4, 7]. Taken together the studies of Wearmouth et al. [3] and Reynolds et al. [4] suggest that stochastic decision-making processes and scale-free patterns of behaviour are pervasive, applying across taxa with divergent foraging strategies, ranging from highly mobile pursuit predators to the less mobile ambush predators. Stochastic-decision making processes could be at work in shearwaters. In this respect, flight durations would be regarded as being waiting-to-land durations, and on/in water durations would be regarded as being waiting-to-fly durations. This explanation of our data does, however, go beyond the capabilities of current models which predict that the durations of one kind of waiting will be power-law distributed, whilst the durations of the other kind of waiting will be exponentially distributed [3, 4]. We found that waiting-to-land durations and waiting-to-fly durations are both power-law distributed. Note that the modeling takes no account of physiological constraints such as the need to rest (sit on the water), after a tiring flight or dive for instance.

## Conclusions

A key objective of the emerging discipline of Movement Ecology is to characterize how resting and active periods are interwoven throughout an animals’ daily life. This is potentially challenging because animal activity patterns often appear to be haphazard, idiosyncratic and apparently unpredictable. Here we provide evidence that the active and resting period durations of shearwaters in their natural environment do, in fact, follow universal statistical laws. Distributions of duration are found to have ‘heavy’ power-law tails that lack characteristic scales. These temporal scaling laws could be the parallel in time of the spatially-invariant ‘Lévy flight’ movement patterns that have been observed across taxa.

A key question for subsequent work is identification of the underlying physiological and behavioural mechanisms. It is possible that olfactory-cued foraging and stochastic-decision making processes are both at work; with the former instigating landings and the later initiating take-offs. It is also possible that the floating bouts are akin to the pauses in motion that occur in some invertebrates when ‘idling’ in the absence of external stimuli. Pause durations tend to be 3/2 power-law distributed [5].

But whatever the mechanism, going forward, we can be more ambitious and expect more from Lévy statistics than just descriptions of movement patterns. Early work was exclusively focused on Lévy walks as models of movement pattern and led to the ‘Lévy flight foraging hypothesis’. This hypothesis has shaped much of subsequent research but as our study makes clear it does not encompass the full gamut of Lévy patterns at work in the biological world. What is required now is a more general form of Lévy theory, one which can account for the ubiquity of Lévy statistics in both spatial and temporal patterns, and for their occurrence in situations that are incompatible with the assumptions underlying the Lévy flight foraging hypothesis [28].

## Methods

### Study areas and species

The Ss and the Cs are two closely related species breeding in the Mediterranean and in the North Atlantic Ocean, respectively. Fieldwork on Ss was carried out in 2008 during both incubation and chick-rearing on Linosa island (Sicily; 35°52’N 12°52’E). 7 Cs were tagged at Berlengas (continental Portugal; 39°24’N 9°30’E) and 8 Cs were tagged at Corvo (Açores; 39°42’N 31°6’W) in 2007 during both incubation and chick-rearing. We caught a total of 25 adult Ss (11 M, 12 F, 2 indeterminate) and 66 adult Cs (39 M, 27 F). During incubation, shearwaters spend several days incubating the egg until their partner, which is foraging at sea, comes back for the changeover. Foraging trips last on average (and SD) 8.43 ± 1.53 days in Linosa Ss [42] and 9.20 ± 6.80 days in Cs [43]. During chick-rearing adults leave the chick alone and return for a daily feed at night. [44] In this phase, foraging trips are on average shorter (Ss; 3.89 ± 0.6, [42]; Cs; 2.09 ± 0.5, [43]) than the ones performed during incubation. During chick-rearing, breeders perform a dual foraging strategy where several short trips, which last about 1–4 days and are mostly for chick provisioning, alternate with longer trips for self-provisioning [44, 45]. Breeders were equipped with compass loggers, a direction and temperature recorder by Earth & Ocean Technologies (Compass-Tlog, Kiel, Germany). Devices were set to collect changes in two bearings (north and east) and temperature information every 5 s. This is a blinded method as the recordings were not made in the presence of an observer. Software MT-Comp v6 (Jensen Software System, Kiel, Germany) was used to reconstruct flight tracks and to interpret behavioural data (diving, flying and resting on the water). For more details regarding tagging and device functioning see Rubolini et al. [46].

### Bird tagging

Birds were caught by hand at the nest both during incubation and chick-rearing. Compass-loggers were attached to the tail feathers using 3-4 strips of Tesa® marine cloth tape (Tesa SE, Hamburg, Germany). All loggers were removed immediately after tagged birds returned from their foraging trip. As recommended by several authors (e.g. [47, 48]), the weight of loggers was kept below 3 % of the birds’ body mass (range: 1.3 – 2.1 %; median = 1.8 %). External devices of this size are considered not to have any detectable influence on the foraging behaviour of Scopoli’s shearwater [49, 50], though they may possibly reduce nestling feeding rate and hamper nestling growth [50]. Furthermore, Paiva et al. [43] reported that there was no significant difference between the weights gained by tagged and non-tagged individuals during both chick provisioning and incubation. Deployment of Compass-loggers took less than 10 min and birds were returned to their nest immediately afterwards.

### Data analysis

Distributions of observed flight durations, on/in water durations and time-intervals between consecutive dives (Fig. 7) were fitted to the 3 most relevant distributions, truncated power-law distributions (1a), exponentially-truncated power-law distributions (1b); and to exponential distributions (1c);

1a $$ {p}_1(l)={N}_1{l}^{-\mu}\kern3em {l}_1\le l\le {l}_2 $$

1b $$ {p}_2(l)={N}_2{l}^{-\mu } \exp \left(-{\lambda}_2l\right) $$

1c $$ {p}_3(l)={N}_3 \exp \left(-{\lambda}_3l\right) $$

where *N*_1_, *N*_2_ and *N*_3_ are normalisation factors which ensure that the distributions sum correctly to unity when integrated over all time-intervals between the lower and upper cut-offs, *l*_*1*_ and *l*_*2*_ (specified below); *μ* is the power-law exponent and, *λ*_2_ and *λ*_3_ are exponential decay rates. Power-laws (Eqn. 1a) with 1 < *μ* ≤ 3 are indicative of Lévy patterns. Exponentially-truncated power-laws (Eqn. 1b) with *μ* > − 1 are, by definition, gamma distributions, a commonly considered form of waiting-time distribution. Exponentially-truncated power-laws with 1 < *μ* ≤ 3 can also be interpreted as being indicative of exponentially truncated Lévy patterns. They can also be regarded as simply providing a continuum of phenomenological, synthetic models that lie between power-laws and exponentials; an approach advocated by Bertrand et al. [51] when attempting to identify the most parsimonious best-fit model. Exponentials (Eqn. 1c) are indicative of Poisson processes. We considered truncated distributions because movements can only take place in finite time (dive times are, for instance, limited by lung capacity) and for this reason only truncated Lévy patterns are possible. When bounded the minimum and maximum truncation scales introduce characteristic scales which make movement patterns scale-finite. But unlike other finite-scale movement patterns variability around the characteristic scales is huge and self-similar, and so bears the hallmark of Lévy patterns. Note that also when Lévy patterns are truncated, the Lévy exponent, *μ*, is not constrained to take values exceeding *1* as the distributions can be normalized with probabilities summing correctly to unity when. *μ < 1.*

Fits to data were obtained using maximum likelihood methods [10, 52] with no restrictions on the values in *μ* when fitting truncated power-law, and exponentially-truncated power-laws. The lower cut-off was taken to be 60 s for resting and flight durations, and 600 s for inter-dive durations, which from visual inspection of the distributions marked the starts of the tails of the distribution functions, and in all cases the upper cut-off was, following Humphries et al. [29], taken to be longest duration/interval in the record. Analysis outcomes do not change significantly when the lower cut-offs were taken to be 30 s and 300 s indicating that our results are robust with respect to the choices for the lower cuts. Our choice for the lower cut-offs thereby ensure statistical consistency of the fitted model.

The best model distribution was identified using the Akaike information criterion [53]. Following Clauset et al. [54] the absolute goodness-of-fit of the best-fit model distribution was quantified by *P*-value. If the *P*-value is large, then the difference between the empirical data and the model distribution can be attributed to statistical fluctuations alone; if it is small, then the model distribution is not a plausible fit to the data. Following Clauset et al. [52] we make the relatively conservative choice and reject the model distribution of interest if *P* ≤ 0.1, otherwise it is accepted as being plausible.

Where there was data for multiple trips for a particular bird only data from the first trip was analysed so that individual birds did not have to be treated as random factors in our analyses. We only analysed data collected during the daytime between 5 am and 8 pm and excluded data collected at night when birds spent most of their time resting or “rafting” (a social behaviour which mainly occurs in front of the colony before coming in) and rarely dive [46].

## Abbreviations

Cs, stands for Cory’s shearwater *C. borealis.*; LW, stands for Lévy walks; Ss, stands for Scopoli’s shearwater *Calonectris diomedea*
